# Long-Term Results of a Comparison Between 15 × 2.633 Gy and 20 × 2.0 Gy for Malignant Spinal Cord Compression in Patients with Longer Expected Survival Times

**DOI:** 10.3390/jcm15114328

**Published:** 2026-06-03

**Authors:** Dirk Rades, Christian Staackmann, Darejan Lomidze, Barbara Segedin, Blaz Groselj, Fernando Lopez Campos, Arturo Navarro-Martin, Jon Cacicedo

**Affiliations:** 1 Department of Radiation Oncology, University Medical Center Schleswig-Holstein, Campus Lübeck, 23538 Lübeck, Germany; christian.staackmann@uksh.de; 2 Radiation Oncology Department, Tbilisi State Medical University and Ingorokva High Medical Technology University Clinic, Tbilisi 0177, Georgia; dlomidze@hotmail.com; 3 Department of Radiotherapy, Institute of Oncology Ljubljana and Faculty of Medicine, University of Ljubljana, 1000 Ljubljana, Slovenia; bsegedin@onko-i.si (B.S.); bgroselj@onko-i.si (B.G.); 4 Department of Radiation Oncology, University Hospital Ramón y Cajal, 28034 Madrid, Spain; flcampos@salud.madrid.org; 5 Department of Radiation Oncology, Clínic Barcelona Villarroel 170, 08036 Barcelona, Spain; anavarroma@clinic.cat; 6 Department of Radiation Oncology, Cruces University Hospital and Biobizkaia Health Research Institute, 48903 Barakaldo, Spain; jon.cacicedofernandezbobadilla@osakidetza.eus

**Keywords:** malignant spinal cord compression, longer expected survival time, radiation therapy alone, higher total doses, long-term results

## Abstract

**Background/Objectives:** A considerable number of patients with malignant spinal cord compression (MSCC) and a longer expected lifespan do not receive upfront surgery but radiation therapy alone. These patients were suggested to benefit from radiation programs with total doses > 30 Gy in terms of better local progression-free survival (LPFS). A previous study compared such regimens, namely 15 × 2.633 Gy over three weeks (34 patients, prospective cohort) and 20 × 2.0 Gy over four weeks (239 patients, control), using a propensity score-adjusted approach. Both regimens were associated with similar rates of overall survival (OS) and LPFS. However, follow-up was limited to 12 months. For long-term survivors, a longer period of follow-up would be desirable. Therefore, the present study was initiated. **Methods:** Retrospective collection of additional data enabled us to provide OS- and LPFS-rates at 36 months following radiation therapy. **Results:** In the prospective cohort, 36-month rates of OS and LPFS were 27.0% and 89.7%, respectively. After application of the propensity score-adjusted Cox regression model, 36-month OS-rates (HR 1.454; 95% CI 0.748–2.828; *p* = 0.270) and LPFS-rates (HR 0.311; 95% CI 0.041–2.352; *p* = 0.258) appeared not considerably different. Late radiation myelopathy and pathologic vertebral fractures were not identified. **Conclusions:** The results of the current study suggest that the role of 15 × 2.633 Gy should be further investigated in selected patients with MSCC, particularly when considering its shorter overall treatment time in comparison to 20 × 2.0 Gy. Overall, our findings are hypothesis-generating rather than confirmatory.

## 1. Introduction

Metastatic lesions of the spine can lead to malignant spinal cord compression (MSCC) [1,2], associated with onerous symptoms including bone pain and neurologic deficits [1,2]. Until 2005, radiation therapy alone was the standard treatment for MSCC. Since a randomized trial suggested that outcomes can be improved by upfront decompressive surgery in selected patients, surgery significantly gained popularity [3]. In that trial, patients were required to have a good performance status and at least an intermediate survival prognosis. Moreover, patients with spinal cord compression from myeloma or lymphoma were not included. The same applied if paraplegia lasted longer than 48 h or MSCC was not restricted to one spinal region [3,4]. Nowadays, patients with limited survival times are mostly treated with radiation therapy alone (supplemented by corticosteroids) [1,2]. However, patients with longer expected survival who meet the criteria for upfront surgery according to the randomized trial from 2005, may also be treated with radiation therapy alone [3]. This group includes patients with a high comorbidity index and very elderly patients but also patients who refuse to undergo surgery. Moreover, since MSCC requires a prompt start of treatment, radiation therapy is the preferred treatment if surgery cannot be performed within an appropriate time frame [5,6]. The majority of patients assigned to radiation therapy are treated with conventional external-beam radiotherapy (EBRT) rather than stereotactic body radiation therapy (SBRT) [7,8,9,10,11,12,13,14].

When a patient is selected for EBRT, radiation oncologists can choose between different regimens, including single-fraction (e.g., 1 × 8.0 Gy), shorter-course multi-fraction (e.g., 5 × 4.0 Gy or 5 × 5.0 Gy), and longer-course multi-fraction treatment (e.g., 10 × 3.0 Gy or 20 × 2.0 Gy) [15,16,17,18,19]. Longer-course regimens were reported to reduce the rate of in-field recurrences of MSCC when compared to shorter-course or single-fraction treatment [17,18,19]. Since the risk of experiencing an in-field recurrence increases with survival time, the endpoint local progression-free survival (LPFS) is of particular interest for long-term survivors. Ten times 3.0 Gy is the most common longer-course regimen used for MSCC in general. In a retrospective study of patients with MSCC and longer expected survival times, 191 patients receiving 10 × 3.0 Gy were matched for ten patient- or disease-related characteristics to 191 patients irradiated with 15 × 2.5 Gy or 20 × 2.0 Gy [20]. The 2-year LPFS rates were 68% and 90%, respectively, in favor of the higher dose regimens. The difference in LPFS was significant on univariate and multivariate analyses. These findings led to a prospective RAMSES-01 trial that investigated the value of high-precision treatment with 15 × 2.633 Gy or 18 × 2.333 Gy [21]. When compared to a historical control group treated with 10 × 3.0 Gy, patients of the RAMSES-01 cohort experienced significantly better LPFS after 12 months. Subsequently, an additional study was performed that compared 15 × 2.633 Gy over three weeks to 20 × 2.0 Gy over four weeks after propensity score adjustment [22]. In that study, the 12-month LPFS rates were 98.1% and 91.6%, respectively. Since the meaning of LPFS increases with lifetime, it would be interesting to see the results after a longer period of follow-up. Therefore, the present study was performed. Additional data were collected for the patients of the RAMSES-01 trial, and the follow-up was no longer censored after 12 months in the control group (as done in the original study). Due to these measures, 15 × 2.633 Gy and 20 × 2.0 Gy could be compared for overall survival (OS) and LPFS at 36 months following radiation therapy. The long-term results of the original RAMSES-01 trial have been recently published; 15 × 2.633 Gy or 18 × 2.333 Gy resulted in significantly better LPFS up to 3 years after completion of radiotherapy [23]. To better identify the potential value of the regimens used in the RAMSES-01 trial, the present study was performed. Since the previous study [23] and the present study were different regarding the dose-fractionation regimen of the control group (10 × 3.0 Gy vs. 20 × 2.0 Gy), it appeared not reasonable to combine both analyses in a single study.

## 2. Materials and Methods

This multi-center study compared 15 × 2.633 Gy and 20 × 2.0 Gy with respect to OS and LPFS. Other objectives of the original RAMSES-01 trial, such as effect of radiotherapy on motor function and ambulatory status and effect of radiotherapy on sensory function, were not evaluated in the present study, because the status after 36 months was expected to be the same when compared to the status at 12 months after radiotherapy. The current study included 34 patients (10 women and 24 men, median age = 67.5 years) from the prospective RAMSES-01 trial who were treated with 15 fractions over three weeks of highly conformal radiation therapy for MSCC between August 2019 and December 2021 [21]. Thirty-three of these patients received 15 × 2.633 Gy as planned, which represented an equivalent dose in 2 Gy fractions (EQD2) of 41.58 Gy when applying an α/β ratio of 10.0 Gy for tumor cell kill [24]. One patient considered an absolute emergency started with one fraction of 3.0 Gy. To achieve an EQD2 very similar to that of 15 × 2.633 Gy, the 3 Gy fraction was supplemented by 12 fractions of 2.633 and two fractions of 2.333. The cumulative EQD2 was 41.31 Gy (α/β = 10 Gy). Thirty-one patients were treated with volumetric modulated arc therapy and three patients with intensity-modulated radiation therapy. According to the study protocol, the planning target volume was covered by the 95% isodose line and encompassed the vertebrae with MSCC plus cranio-caudal margins of 1 cm. For safety reasons, the relative dose to the spinal cord should not exceed 101.2%, i.e., an EQD2 of 46.6 Gy for radiation myelopathy when applying an α/β ratio of 2.0 Gy) [24,25]. In accordance with the Quantitative Analyses of Normal Tissue Effects in the Clinic (QUANTEC), the mean doses for the heart, lung and esophagus should be kept below 26 Gy, 7 Gy and 34 Gy, respectively [25]. However, the mean heart dose stated in the QUANTEC table was substantially higher than more recently proposed constraints. For example, in a propensity score-matched study from 2026 in patients receiving palliative radiotherapy, a mean heart dose of ≥5 Gy was associated with a significantly worse OS [26]. Therefore, a threshold of 26 Gy appears no longer appropriate and should be replaced by 5 Gy. Specific criteria to be eligible for the RAMSES-01 trial included malignant compression located in thoracic or lumbar spinal cord, at least mild motor deficits of one or both lower extremities existing for a maximum of 30 days prior to radiation therapy, confirmation of the diagnosis by magnetic resonance imaging (preferred) or computed tomography, and longer expected survival according to a validated survival score [21]. Upfront presentation to a neurosurgeon or orthopedic surgeon and concurrent treatment with corticosteroids were strongly recommended. The distribution of patient- and disease-related characteristics was indicated before [21]. Ethics approval was obtained by the committee at the University of Lübeck for the RAMSES-01 trial (file 18-360), the previous secondary analysis with a maximum follow-up of 12 months (file 2024-491), and for the present study (2025-404).

For the 34 patients previously included in the RAMSES-01 trial, OS- and LPFS-rates at 36 months were calculated. Moreover, these patients were compared to a control group of 239 patients from an existing anonymized database. Patients of the control group met the same inclusion criteria as required for the RAMSES-01 trial. They were irradiated with 20 × 2.0 Gy over four weeks. The corresponding EQD2 was 40.0 Gy (α/β = 10 Gy) for tumor cell kill and 40.0 Gy (α/β = 2 Gy) for myelopathy. Investigated endpoints included OS and LPFS at 36 months after completion of radiation therapy. LPFS was defined as freedom from progression of motor weakness during radiation therapy and freedom from a recurrence of MSCC in the previous planning target volume (in-field recurrence) associated with motor weakness in one or both legs. For the RAMSES-01 trial, regular follow-up visits including clinical assessment of Karnofsky performance score, ambulatory status, motor function, sensory function, sphincter dysfunction, vertebral pain (pain score, intake of analgesics), distress, and toxicity were performed at the end of radiation therapy plus one, three, six, nine and 12 months after its completion [21]. In case of new or progressive neurologic symptoms or vertebral pain, spinal imaging was performed at any time during the follow-up to identify an in-field recurrence of MSCC, radiation-related myelopathy, or a pathological vertebral fracture immediately. The additional data collected for the present study were not obtained from a structured schedule of follow-up. Clinical assessment supplemented by diagnostic imaging of the spine was performed only if the patient experienced progressive existing or new vertebral pain or neurologic symptoms. This procedure was also used for the retrospective control group during the entire follow-up.

### Statistical Considerations

For the patients of the RAMSES-01 trial, OS- and LPFS-rates at 36 months were calculated using the Kaplan–Meier method, referenced from the last day of radiation therapy. Subsequently, these patients were compared to the patients of the control group with respect to OS and LPFS. Initially, univariate analyses (Kaplan–Meier method plus log rank test) and multivariate analyses (Cox regression model) of unadjusted data were performed. To reduce the risk of biases caused by differences regarding the distribution of ten patient- and disease-related characteristics, a propensity score adjustment was performed. The procedure was the same as in the previous secondary analysis of the RAMSES-01 trial limited to a follow-up of 12 months [22]. Thus, characteristics used for the propensity score adjustment included age at the time of radiation therapy, gender, interval from initial tumor diagnosis and MSCC, visceral or additional bone metastases outside the area of MSCC, primary tumor type, dynamic (time developing) motor weakness, ability to walk prior to radiation therapy, number of affected vertebrae, and performance status. Since the information for all of these characteristics was available in both groups, there were no missing data during the propensity score modeling. For better comparability, OS and LPFS and curves were censored at 36 months.

After estimating propensity scores for receipt of radiation therapy, a Cox proportional-hazards model with radiation therapy as the exposure and the logit-transformed propensity score as an adjustment term was fitted. To avoid assuming a strictly linear association between the propensity score and the outcome, the logit propensity score was modeled with a restricted cubic spline with five knots [27]. In brief, this approach fits a smooth curve made up of connected cubic segments; the curve is allowed to change its shape at a small number of prespecified knot locations and is constrained to be linear at the extremes. The knot locations were chosen using five equally spaced percentiles of the logit propensity-score distribution, so that knots are placed at data driven cut points across the observed range (i.e., each segment covers a similar proportion of patients).

This approach was already pre-specified in the study protocol of the original RAMSES-01 trial [21]. The statistical analyses of this additional study were performed with version 9.4 of the SAS software (SAS, Cary, NC, USA). For all statistical analyses, a *p*-value of <0.05 was considered to indicate significance.

## 3. Results

**Figure 1:**
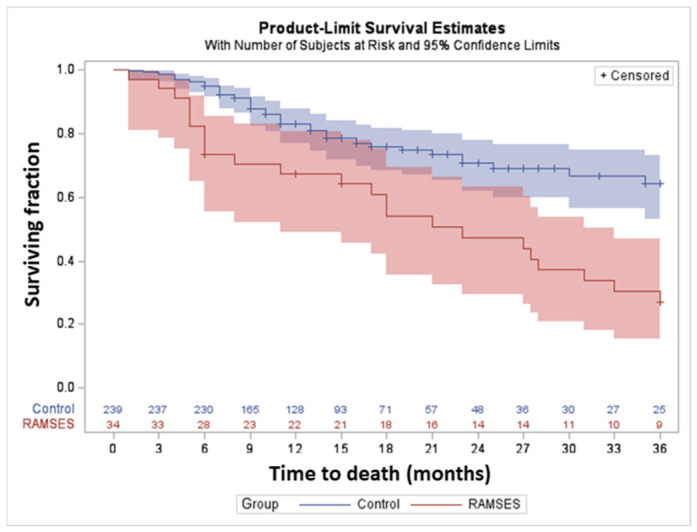
Overall survival: Kaplan–Meier estimates (censored at 36 months).

Twenty-three of the 34 patients included in the prospective part of the RAMSES-01 trial survived longer than 12 months after completion of their radiation therapy. For the present study, additional follow-up data regarding LPFS, OS, and toxicity were obtained for 21 of these patients. These data were from follow-up visits at the treating department of radiation oncology or other departments of the corresponding medical facilities. In the 21 patients, the last documented follow-up visit took place after a median of 31 months (range 15–61 months). In the control group, the follow-up period was extended beyond 12 months for 119 patients. Overall median follow-up times were 17.5 months (range 1–61 months) in patients of the prospective part of the RAMSES-01 trial and 12.0 months (1–85 months) in the control group. A significant disparity was observed between unadjusted groups regarding the OS-rates at 36 months. Until this timepoint, 23 patients died in the RAMSES-01 cohort and 50 patients in the control group. The corresponding OS-rates were 27.0% (12.9–43.3%) and 64.2% (53.2–73.3%), respectively (*p* < 0.001, unadjusted univariate analysis) (Figure 1). In the unadjusted Cox regression analysis, the difference remained significant [hazard ratio (HR) 2.552; 95% confidence interval (CI) 1.545–4.214; *p* < 0.001)].

**Figure 2:**
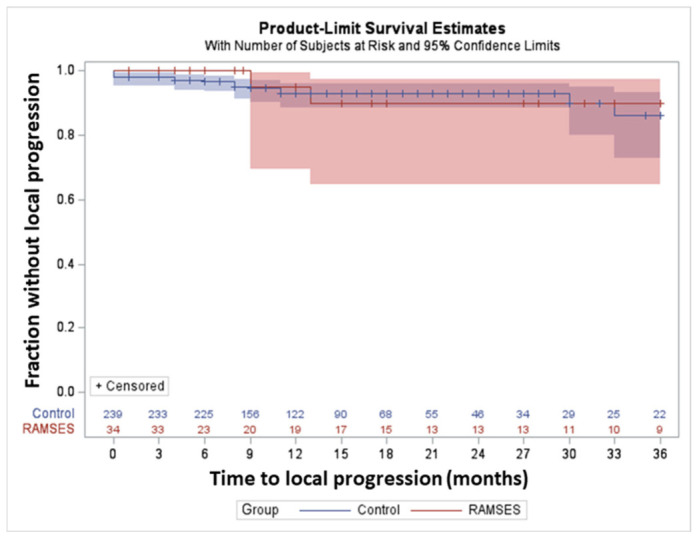
Local progression-free survival: Kaplan–Meier estimates (censored at 36 months).

Two patients of the phase 2 cohort developed local progression of MSCC after nine and 13 months, respectively. In the control group, 16 patients experienced local progression within 36 months and additional three patients after 38, 39, and 42 months, respectively. In the prospective cohort and the control group, the LPFS-rates at 36 months were 89.7% (64.8–97.3%) and 86.2% (72.9–93.3%), respectively (*p* = 0.762, unadjusted univariate analysis) (Figure 2). In the unadjusted Cox regression analysis, the difference was also not significant (HR 0.796; 95% CI 0.180–3.515; *p* = 0.763).

**Figure 3:**
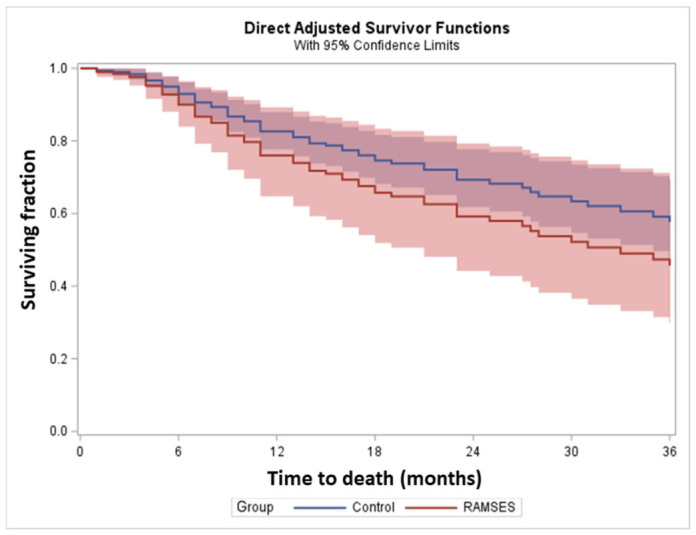
Estimated overall survival after propensity score adjustment (censored at 36 months).

**Figure 4:**
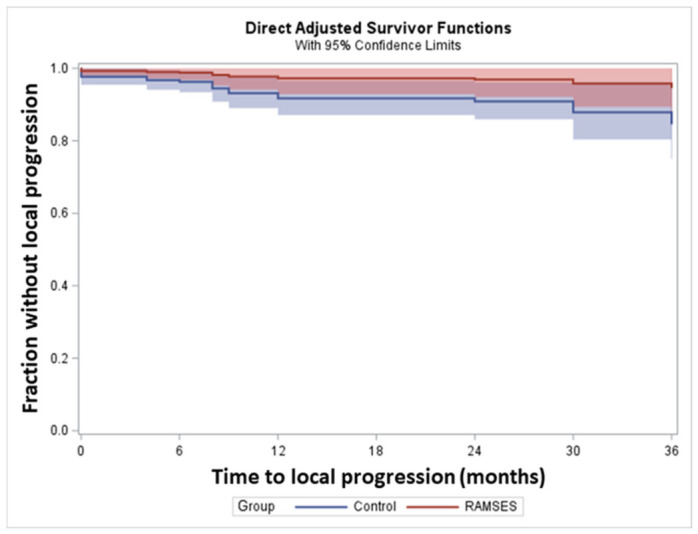
Estimated local progression-free survival after propensity score adjustment (censored at 36 months).

When applying the propensity score-adjusted Cox regression model, 36-month OS-rates (HR 1.454; 95% CI 0.748–2.828; *p* = 0.270, Figure 3) and 36-month LPFS-rates (HR 0.311; 95% CI 0.041–2.352; *p* = 0.258, Figure 4) appeared not considerably different after 15 × 2.633 Gy and 20 × 2.0 Gy. Given the limitation that the follow-up was mainly not structured, myelopathy or pathologic vertebral fractures were not identified.

## 4. Discussion

Most patients presenting with MSCC receive radiation therapy, either alone or following surgical intervention [1,2,28]. When considering the inclusion criteria of a previous randomized trial comparing radiotherapy with and without upfront decompressive surgery, radiation therapy alone is preferentially administered to patients with a poor general condition and patients with a limited estimated survival time [3]. However, patients considered longer-term survivors who meet the above-mentioned criteria may also receive radiation therapy alone. This accounts particularly for patients in whom surgery cannot be performed as an emergency treatment within the required time frame [5,6]. The majority of patients assigned to radiation therapy alone receive conventional EBRT. For patients with an estimated survival time of more than six months, radiation therapy with doses of at least 30 Gy given over two or more weeks appears appropriate [17,18,19]. Such longer-course treatments result in better LPFS when compared to single-fraction or short-course multi-fraction irradiation. Moreover, a previous matched-pair study of patients with MSCC and longer expected survival times suggested that total doses beyond 30 Gy (37.5 Gy in 15 fractions or 40.0 Gy in 20 fractions) resulted in significantly better 1-year and 2-year LPFS rates than 10 × 3.0 Gy [20]. The EQD2 of 37.5 Gy in 15 fractions was 39.06 Gy (α/β = 10 Gy), and thus similar to 40.0 Gy in 20 fractions (20 × 2.0 Gy) [24]. In the RAMSES-01 trial 15 × 2.633 Gy (EQD2 = 41.58 Gy) and 18 × 2.333 Gy (EQD2 = 43.17 Gy) were compared to 10 × 3.0 Gy [21]. The higher dose regimens led to a significantly better 12-month LPFS. In an additional analysis that compared 15 × 2.633 Gy and 20 × 2.0 Gy, the 12-month LPFS-rates were suggested to be not significantly different [22]. For long-term survivors, it would be interesting to know the results after a follow-up period beyond 12 months.

The current study provided results up to 36 months following completion of radiation therapy. When comparing unadjusted groups, 36-month LPFS was not significantly different between both groups. However, patients of the control group had a significantly better 36-month OS on both univariate and Cox regression analyses. This finding could be explained by the differences regarding the distribution of patient- and disease-related characteristics. In the prospective cohort, a higher proportion of the patients had other bone or visceral metastases, were elderly patients, or presented with a worse performance status [22]. These differences support the need for a propensity score adjustment considering several patient- and disease-related characteristics. After propensity score adjustment, both OS and LPFS at 36 months were not significantly different.

This can be explained by the similar EQD2s for tumor cell kill [24]. However, due to the very low number of local progression events and the resulting wide confidence intervals, the present study is underpowered to exclude clinically relevant differences in either direction. Consequently, the findings are to be interpreted with caution and cannot be used to formally demonstrate equivalence or non-inferiority between the two treatment schedules. Moreover, in both groups, relevant late radiation-related toxicities were not identified. Using 15 × 2.633 Gy would mean that the patients need fewer treatment sessions, which can be considered a benefit, particularly if the patients have to travel a long distance to reach the radiation oncology department or are required to undergo their treatment as in-patients. These considerations may also apply to patients with intradural metastatic disease, whose therapeutic management often does not substantially differ from that of pure epidural MSCC in selected clinical scenarios [29,30].

However, any potential clinical equivalence between the regimens cannot be concluded from the present data and would require confirmation in adequately powered prospective trials. The limitations of the current study are given in the corresponding paragraph below.

### Limitations of the Current Study

A significant limitation of the current study is its non-randomized nature plus its hybrid prospective–retrospective design. The experimental cohort originated from a prospective trial, whereas the comparator arm came from a retrospective database. This introduces unavoidable ascertainment and surveillance bias. The prospective arm had structured follow-up during the first year, whereas the retrospective cohort relied on symptom-triggered imaging throughout follow-up. Consequently, local failures in the retrospective arm might have been missed, especially asymptomatic or mildly symptomatic recurrences. Therefore, the LPFS in the control group might have appeared better than it really was. This limitation also applied to data obtained from the patients of the RAMSES-01 trial after 12 months of follow-up. After completion of the original RAMSES-01 trial, these patients were irregularly seen by radiation oncologists or other physicians from the institutions participating in the trial only when they developed new or progressive neurologic symptoms. Moreover, the hybrid design led to a difference with respect to median follow-up times. In contrast to the prospective cohort, follow-up could not be updated in the control group, since the retrospective database was anonymized.

The patients of the RAMSES-01 cohort were treated more recently than the patients of the control group and might, therefore, have benefited from advances in systemic treatment leading to a significant improvement in OS [31,32,33,34]. This hypothesis is supported by the fact that unadjusted analysis demonstrated dramatically better OS in the control. Such a large baseline difference strongly suggests fundamentally different patient populations. Propensity adjustment can only account for measured variables.

The fact that the stability of the spine was not monitored prior to, during and following radiotherapy, e.g., by applying the spinal instability neoplastic score (SINS) [35,36], might have introduced a hidden selection bias. The same applied to the fact that the Bilsky score was not available in the control group and, therefore, could not be included in the propensity score-adjusted Cox regression model [37,38,39]. SINS and Bilsky score strongly influence treatment selection, neurologic outcomes, surgical candidacy, and local control. Their absence substantially limits confidence in the adjusted comparisons.

The identification of radiation-related myelopathy and pathologic vertebral fractures was likely be complicated in both groups due to the fact that spinal imaging was performed only in the case of new or progressive pain or neurologic symptoms and not at pre-specified time points. Therefore, the absence of radiation myelopathy or pathologic vertebral fractures cannot be interpreted as evidence of safety. In addition, the sample size in the prospective cohort is too small to meaningfully evaluate rare late toxicities such as radiation myelopathy. Moreover, the imbalance in cohort sizes and the small prospective sample likely created substantial instability in the statistical estimates regarding LPFS, since only two local progression events occurred in the experimental cohort. The resulting confidence intervals are extremely wide, indicating that the study cannot exclude clinically meaningful inferiority or superiority. The study was not prospectively designed as a non-inferiority trial. No non-inferiority margin was pre-specified, and no power calculation for non-inferiority was performed. Therefore, the absence of statistically significant differences must not be interpreted as evidence of equivalence or non-inferiority. Given the small number of LPFS events observed, the study had limited ability to detect anything other than large differences between both regimens, further underscoring its exploratory nature. The fact that the number of LPFS events was comparably small may also be a consequence of the definition of the primary endpoint LPFS. Local progression required recurrence associated with motor deficits. Thus, radiologically identified progression without overt motor deterioration were not counted as an event. Focusing on symptomatic recurrences of MSCC appears reasonable for patients with a limited life expectancy. However, in patients with more favorable survival prognoses, local control endpoints should be imaging-based rather than purely neurologic-function-based.

All patients previously included in the RAMSES-01 trial received highly conformal radiation therapy with volumetric modulated arc therapy or intensity-modulated radiation therapy, which was used only for the minority of patients in the control group. The use of highly conformal radiation techniques may have reduced radiation-related toxicity. Outcomes may also have been improved by new developments in diagnostic imaging, treatment planning, immobilization, and supportive care in patients of the RAMSES-01 cohort, who were irradiated more recently than the patients of the control group. It should also be noted that this study did not include patients treated with SBRT, since this technique is preferably used for reirradiation of spinal metastases [40,41,42,43]. SBRT is becoming more popular for selected patients with only one or few spinal metastases leading to MSCC [7,8,9,10,11,12,13,14]. The advantage of SBRT is the combination of high biological effective doses and a short overall treatment time. However, this approach is still controversial for true MSCC associated with motor deficits, because the recommended minimum distance of 3 mm between the metastatic lesion and the spinal cord cannot be achieved [44]. Moreover, SBRT bears a certain risk of post-treatment vertebral fractures and adjacent-level tumor progression [7,23,45]. Thus, external-beam radiotherapy still is the most common type of radiotherapy used for MSCC with motor deficits.

For patients who need immediate systemic treatment radiotherapy with 15 × 2.633 Gy over three weeks may not be appropriate. For many of these patients, radiation and medical oncologists need to strike a balance between potentially better local control of MSCC that can be achieved with longer-course radiotherapy over three weeks and an earlier start of systemic treatment. The decision likely depends on several factors including the extension of metastatic disease. For patients with very few bone metastases and no visceral lesions, 15 × 2.633 Gy may be an option, whereas for patients with multiple bone and/or visceral metastases early initiation of systemic treatment is very important. In recent years, the concurrent administration of radiotherapy and newer systemic agents such as cyclin-dependent kinase (CDK) inhibitors has become increasingly common in metastatic disease management [46]. While this combination is generally feasible and safe, the risk profile may significantly depend on the irradiated vertebral level. In particular, irradiation of thoracic vertebral segments during CDK inhibitor therapy may substantially increase the risk of pulmonary toxicity. In these cases, the sequencing of systemic treatment and radiotherapy should be carefully individualized through close multidisciplinary discussion between medical and radiation oncologists. Radiotherapy with a shorter overall treatment time may also help reduce the potential overlap-related toxicities and facilitate safer treatment integration.

The findings of the current study may not be generalized to patients receiving SBRT or upfront spinal surgery, particularly in case of more unstable lesions [47,48,49,50,51,52,53,54,55,56]. Current management increasingly incorporates surgery, with or without postoperative SBRT, or separation surgery paradigms, especially in patients with a favorable prognosis. Thus, the applicability of the results of this study is limited to patients unable or not willing to receive surgery or SBRT. Moreover, the results may be relevant for countries with limited availability of neurosurgery and/or stereotactic radiotherapy.

## 5. Conclusions

The results of this study suggest that the potential value of radiotherapy with 15 × 2.633 Gy should be further investigated in selected patients with MSCC, particularly when considering its shorter overall treatment time in comparison to 20 × 2.0 Gy. However, when interpreting the results, the limitations of the current study must be considered, particularly its non-randomized design and the absence of a prospectively defined non-inferiority margin and associated power calculation, plus the retrospective nature of the control group lacking a standardized follow-up. Moreover, our results may not be generalized to patients assigned to SBRT or upfront surgery. An adequately powered prospective randomized trial would be required to reliably assess comparative effectiveness of 15 × 2.633 Gy for definitive radiation therapy of MSCC in patients with favorable survival prognoses. Such a trial may be difficult to perform, because the majority of potentially suitable patients likely receive upfront surgery. Overall, our findings are fundamentally hypothesis-generating rather than confirmatory or even practice-changing.

## Data Availability

The RAMSES-01 trial has been registered at clinicaltrials.gov (identifier NCT04043156). Other results of the current study may not be shared due to data protection regulations.

## Abbreviations

The following abbreviations are used in this manuscript:

EBRT	External-Beam Radiotherapy
EQD2	Equivalent Dose in 2-Gy Fractions
LPFS	Local Progression-Free Survival
MSCC	Malignant Spinal Cord Compression
OS	Overall Survival
SBRT	Stereotactic Body Radiation Therapy
SINS	Spinal Instability Neoplastic Score
